# Biomechanical analysis of limits of stability using computerized posturography: correlations with functional mobility in elderly individuals with hip osteoarthritis – a cross-sectional study

**DOI:** 10.3389/fbioe.2024.1440393

**Published:** 2024-11-25

**Authors:** Khalid A. Alahmari, Ravi Shankar Reddy

**Affiliations:** Biomechanics and Research Lab, Program of Physical Therapy, Department of Medical Rehabilitation Sciences, College of Applied Medical Sciences, King Khalid University, Abha, Saudi Arabia

**Keywords:** hip osteoarthritis, limits of stability, functional mobility, elderly, posturography

## Abstract

**Background:**

Hip osteoarthritis (OA) is a degenerative joint disease that predominantly affects the elderly, causing significant morbidity due to joint pain, stiffness, and loss of function. This study aimed to assess the limits of stability (LOS) using computerized posturography and evaluate the correlations with functional mobility in elderly individuals with hip OA.

**Methods:**

This cross-sectional study included elderly individuals aged 65 years and above with a clinical diagnosis of hip OA and age-matched asymptomatic controls. The LOS was measured using a computerized dynamic posturography system, which quantified the maximum distance and angle participants could shift their center of gravity without losing balance. Functional mobility was assessed using the Timed Up and Go (TUG) test, which measures the time taken for participants to stand up, walk 3 m, turn around, walk back, and sit down.

**Results:**

The study included 86 elderly individuals with hip OA and 86 age-matched asymptomatic controls. LOS assessments showed that individuals with hip OA had significantly lower stability scores across all directions compared to controls (p < 0.001). TUG test times were significantly slower for the OA group (10.50 ± 2.20 s) compared to controls (8.70 ± 2.00 s, p < 0.001). Positive correlations were found between LOS and functional mobility (r = 0.50, p = 0.009). Moderation analysis revealed that age and duration of OA significantly influenced the relationship between stability and mobility.

**Conclusion:**

Hip OA significantly impacts stability and functional mobility in elderly individuals. Enhanced stability is associated with improved mobility, and demographic and clinical variables such as age and duration of OA play crucial roles in these relationships. These findings underscore the importance of targeted therapeutic interventions to improve stability and mobility in this population.

## 1 Introduction

Hip osteoarthritis (OA) is a degenerative joint disease that predominantly affects the elderly, leading to significant morbidity due to joint pain, stiffness, and loss of function (Abramoff and Caldera, 2020). Characterized by the gradual deterioration of cartilage and changes in the periarticular bone, hip OA is a major cause of impaired mobility and reduced quality of life among older adults (Murphy et al., 2016). It remains a public health challenge due to its increasing prevalence in the aging population and the substantial personal and socioeconomic burdens it imposes (Egerton et al., 2018). Although the pathogenesis of hip OA involves biomechanical and inflammatory processes that alter joint structure and function, the specific contributions of these changes to the functional impairments observed in individuals remain to be fully elucidated (Litwic et al., 2013).

The concept of “limits of stability” (LOS) is critical in understanding how individuals maintain balance when their center of gravity approaches the boundaries of their base of support (Nolff et al., 2024). LOS represents the maximum angle or distance one can tilt or move without losing balance, stepping, or falling (Cella et al., 2020). In patients with hip OA, LOS may be significantly compromised due to joint instability, pain, muscular weakness, and compensatory gait adaptations (Boekesteijn et al., 2021). These biomechanical constraints not only limit movement but also contribute to an increased risk of falls (Sturnieks, 2021). Thus, evaluating LOS in the context of hip OA can provide insights into the extent of mobility limitations and the effectiveness of various therapeutic interventions aimed at restoring balance and preventing falls (Grabiner and Kaufman, 2021).

Functional mobility, defined as the ability to move independently to carry out activities of daily living, is a key determinant of autonomy and quality of life in the elderly (Tornero-Quiñones et al., 2020). In the context of hip OA, functional mobility is often compromised, with affected individuals experiencing difficulties in walking, climbing stairs, and performing other activities that require joint movement and stability (Do and Yim, 2020). The deterioration in functional mobility is primarily due to pain, muscle weakness, and joint stiffness, which directly affect the stability and biomechanical efficiency of the hip (Boekesteijn et al., 2021). Consequently, there is a profound need to explore the relationships between LOS, joint stability, and functional mobility to develop targeted strategies that can enhance the quality for those afflicted.

Despite extensive research on hip OA, there remains a significant gap in understanding how demographic and clinical variables modulate the relationship between stability and functional mobility in this population (Laitner et al., 2021). Previous studies have primarily focused on the impact of OA on overall physical function with less emphasis on specific biomechanical measurements such as LOS and their correlation with everyday functional tasks (Wilson and Kobsar, 2021). Moreover, the influence of factors such as age, disease duration, and body mass index on these relationships has not been adequately addressed (Batushansky et al., 2022). This lack of detailed knowledge hampers the development of customized treatment and rehabilitation protocols that address the specific needs of different patient subgroups (Brembo, 2020). Therefore, a comprehensive study integrating biomechanical, clinical, and demographic variables is essential to elucidate these complex interactions and guide clinical practice.

This study aims to fill these research gaps by 1) comparing the limits of stability and functional mobility in elderly individuals with hip osteoarthritis *versus* age-matched asymptomatic controls; 2) evaluating the association between stability limits and functional mobility within the hip OA group; and 3) conducting a moderation analysis to determine how age, gender, body mass index, and duration of osteoarthritis diagnosis influence these relationships. The hypotheses are that stability limits will be significantly lower, functional mobility will be more impaired in individuals with hip OA compared to controls, and the severity of these impairments will be moderated by demographic and clinical variables. These objectives will provide a comprehensive understanding of the biomechanical deficits in hip OA and support the development of targeted interventions to improve stability and mobility in this vulnerable population.

## 2 Materials and methods

### 2.1 Study design, settings, and duration

This descriptive cross-sectional study took place from June 2022 to May 2023 in the Physical Therapy Department of King Khalid University Hospital, a tertiary care facility located in Abha, Saudi Arabia, which focuses on geriatric and orthopedic care. The participants were enlisted from the outpatient orthopedic clinic and the geriatric department within the hospital. The research protocol was reviewed and approved by the King Khalid University Institutional Review Board (approval number REC# 456–872) and conformed to the ethical guidelines outlined in the Declaration of Helsinki. All participants provided written informed consent before inclusion in the study.

### 2.2 Participants

The study involved elderly individuals aged 65 and above, both with and without hip osteoarthritis, who were recruited from outpatient orthopedic clinics in Abha, Saudi Arabia. The inclusion criteria for the osteoarthritis group were a clinical diagnosis of hip osteoarthritis based on the American College of Rheumatology criteria, the ability to walk independently, and willingness to participate in the study (van Doormaal et al., 2020). Age-matched asymptomatic controls were selected based on the absence of hip pain, osteoarthritis, or other musculoskeletal disorders. Participants who were able to understand and follow study instructions and who provided written informed consent were also included. Exclusion criteria for both groups were comprehensive to ensure the validity of the study. Individuals were excluded if they had other musculoskeletal disorders that could affect balance and mobility, such as severe knee osteoarthritis or lower limb fractures. Neurological conditions which could impair balance, were also grounds for exclusion. Cognitive impairments, determined by a score of less than 24 on the Mini-Mental State Examination (MMSE), were another exclusion criterion, as such impairments could interfere with the ability to understand and perform the study tasks. Participants who had undergone hip replacement surgery or other major lower limb surgeries within the past 6 months were excluded to avoid confounding factors related to recent surgical recovery. Additionally, individuals with severe visual or auditory impairments that could affect their ability to follow instructions or perform the tasks were not included. Lastly, any participant with an unstable medical condition or comorbidity that could pose a risk during the study assessments, such as uncontrolled hypertension or severe cardiovascular disease, was excluded.

### 2.3 Limits of stability assessment

**FIGURE 1:**
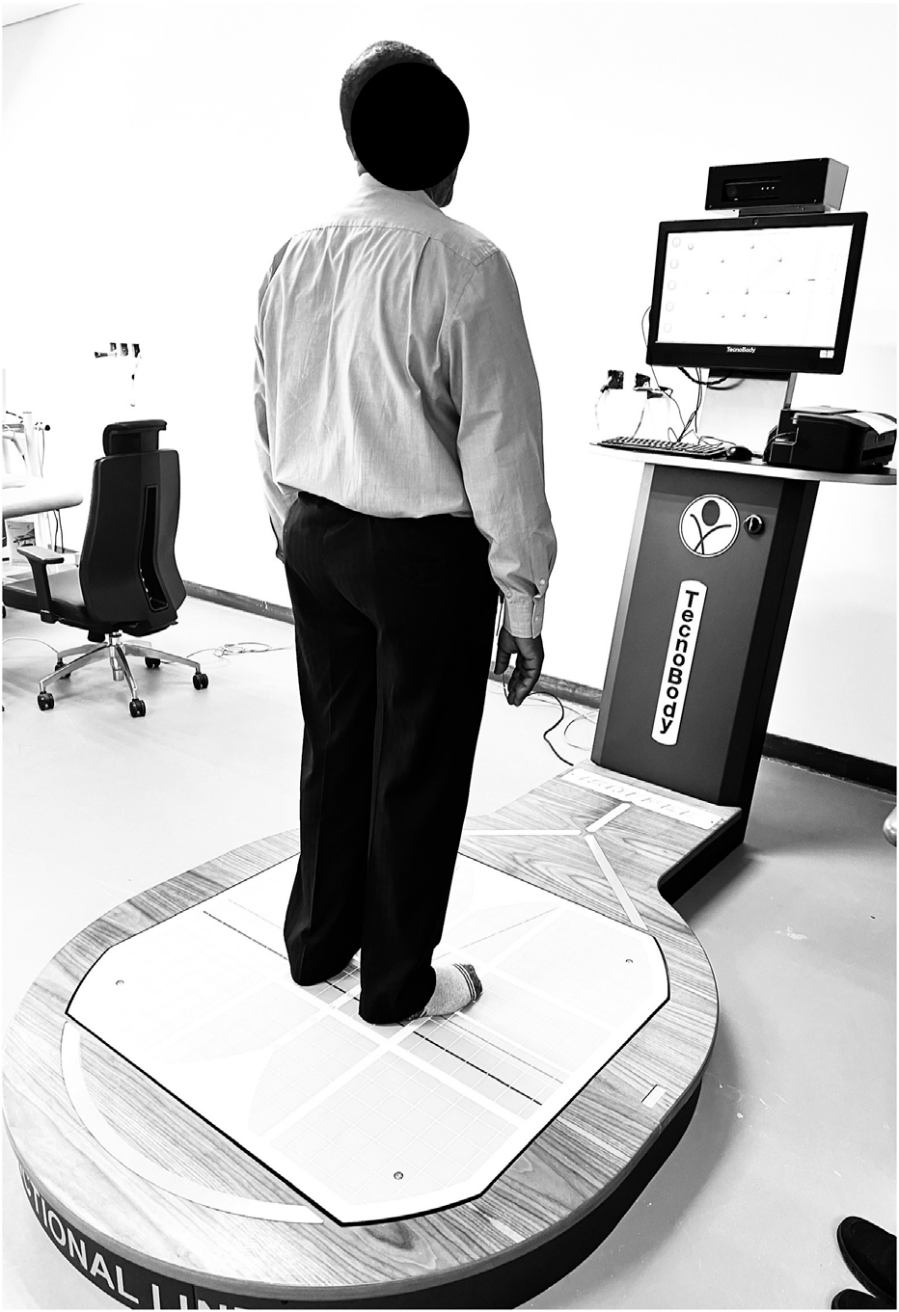
Evaluation of Stability Boundaries in eight directions, illustrated with panel showing the initial starting position and panel displaying the participant reaching a predetermined target, as seen in computerized posturography.

The LOS were assessed using computerized dynamic posturography, which incorporates a stabilometric posture platform, a touch screen, a 3D camera, and dedicated software (Fonseca et al., 2021). This system measures the maximum distance and angle at which participants can shift their center of gravity without losing balance, stepping, or falling (De Jong et al., 2020). The LOS assessments were performed in a calm, well-ventilated setting. Participants stood on the stabilometric force platform with their feet together in a standardized position, facing a screen that displayed targets (Figure 1). These targets appeared randomly in eight directions: forward, backward, left, right, and intermediate diagonal directions (left-forward, right-forward, left-backward, right-backward) (Palm et al., 2014). Participants were instructed to shift their center of mass toward the targets without moving their feet. The posturography system recorded the amount of sway required to reach each target, providing a score based on the precision of movement along the shortest vertical or horizontal path. A score of 100 indicated perfect stability in a given direction, while lower scores reflected greater sway and reduced stability. This methodology ensured that LOS data were collected with high consistency and accuracy, yielding reliable measurements of the participants’ balance and stability capabilities.

### 2.4 Functional mobility assessment

Functional mobility was assessed using the TUG test, which is a widely recognized and reliable measure of functional mobility and fall risk (Bergquist et al., 2020). During the TUG test, participants are timed as they rise from a seated position in a standard armchair, walk 3 m, turn around, walk back to the chair, and sit down again. This evaluation provides a quick and practical measure of the basic mobility skills necessary for daily activities. The test was conducted in a well-lit, unobstructed corridor within the hospital to ensure both safety and accuracy. Participants received clear instructions to perform the task at their usual walking pace, reflecting their typical level of mobility. The time required to complete the TUG test was recorded in seconds using a calibrated stopwatch. Performance on the TUG test serves as a key indicator of functional mobility, with shorter times indicating better mobility (Rose et al., 2002).

### 2.5 Pain and Disability Scores

Pain levels were measured using the Visual Analog Scale (VAS), where participants rated their pain on a scale from 0 (no pain) to 10 (worst pain imaginable). Functional disability was evaluated using the Harris Hip Score, a standardized questionnaire that assesses pain, function, absence of deformity, and range of motion.

### 2.6 Sample size estimation

The sample size for our cross-sectional study was determined using G*Power statistical software, version 3.1.9.4. We targeted an effect size of 0.4 to detect moderate effects of hip osteoarthritis on stability and mobility (Alkhamis et al., 2024). For the limits of stability (LOS), we assumed a mean of 78.00 (SD = 9.00) for the control group and 68.00 (SD = 8.00) for the OA group. For the Timed Up and Go (TUG) test, the assumed means were 8.70 s (SD = 2.00) for the control group and 10.50 s (SD = 2.20) for the OA group. Cohen’s d was computed as the difference in means divided by the pooled standard deviation. For LOS, Cohen’s d was calculated as [(78.00–68.00)/8.53] ≈ 0.50, and for TUG as [(10.50–8.70)/2.10] ≈ 0.85. Calculations aimed for a power of 0.80 and an alpha level of 0.05, indicating that 86 participants per group were required to conduct reliable independent samples t-tests, correlation analyses, and moderation analyses.

### 2.7 Data analysis section

Data processing and analysis were conducted using the Statistical Package for the Social Sciences (SPSS), version 21. Preliminary checks for normality and outliers ensured that the assumptions for parametric statistical testing were met, as indicated by the Shapiro-Wilk tests and acceptable levels of skewness and kurtosis, confirming that data distributions were approximately normal. For Objective 1, we employed independent samples t-tests to compare the LOS and functional mobility between elderly individuals with hip OA and age-matched asymptomatic individuals, with Cohen’s d calculated to assess effect sizes. Objective 2 involved analyzing the relationship between stability limits and functional mobility using Pearson correlation coefficients to explore the strength and direction of their association in the hip OA group. For Objective 3, moderation analysis with multiple regression models tested how demographic and clinical variables such as age, gender, body mass index (BMI), and duration of OA diagnosis might moderate this relationship (Jie et al., 2015). Interaction terms were created and simple slopes analysis was performed for significant interactions, adjusting for multiple comparisons to ensure precise interpretation. Assumptions of multicollinearity, independence of residuals, homoscedasticity, and linearity were thoroughly checked through diagnostic plots and tests, including VIF and Durbin-Watson, to validate the regression models (Srinivasan et al., 2017). All statistical tests were two-tailed with a significance level set at p < 0.05, providing a robust and reliable framework for evaluating the effects of stability and mobility in our study population.

## 3 Results

The demographic and clinical characteristics of the study participants reveal significant differences in Body Mass Index (BMI), Pain, VAS, and Disability Scores between elderly individuals with hip osteoarthritis and asymptomatic controls, while age and gender distribution show no significant differences (Table 1). Specifically, participants with hip osteoarthritis had a higher average BMI (29.13 ± 4.25) compared to controls (27.56 ± 3.89), with a statistically significant difference (p = 0.013). The Hip Osteoarthritis group had an average duration of osteoarthritis diagnosis of 5.25 ± 2.67 years, a mean pain score of 6.54 ± 1.28 on the Visual Analog Scale (VAS), and a mean disability score of 62.35 ± 8.14 based on the Harris Hip Score.

**TABLE 1:** Demographic and clinical characteristics of study participants.

Characteristics	Hip osteoarthritis (n = 86)	Asymptomatic controls (n = 86)	p-value
Age (years)	72.34 ± 5.46	71.87 ± 5.17	0.424
Gender (Male/Female)	41/45	40/46	0.882
Body Mass Index (kg/m^2^)	29.13 ± 4.25	27.56 ± 3.89	0.013*
Duration of OA Diagnosis (years)	5.25 ± 2.67	N/A	N/A
Pain VAS (0–10 scale)	6.54 ± 1.28	-	N/A
Disability Score (Harris Hip Score)	62.35 ± 8.14	-	N/A

OA, osteoarthritis; VAS, visual analog scale.

The analysis presented in Table 2 underscores the marked differences in LOS and functional mobility between elderly individuals with hip osteoarthritis and age-matched asymptomatic controls. Notably, the group with hip osteoarthritis demonstrated significantly lower stability percentages across all directional measures, with the forward direction showing the greatest disparity: 41.20 ± 4.50 for osteoarthritis patients compared to 78.00 ± 9.00 for controls, and a p-value of less than 0.001. Similar significant differences were observed in other directions, including the right-forward direction (68.00 ± 8.00 vs. 88.00 ± 11.00), right (72.00 ± 11.00 vs. 92.00 ± 12.00), and left-forward (88.00 ± 11.00 vs. 97.00 ± 14.00), all with p-values below 0.001. Cohen’s d values indicate moderate to large effect sizes, particularly notable in the right-forward direction (0.50) and total objective measures (0.49). Additionally, functional mobility as assessed by the TUG score showed osteoarthritis patients had significantly slower times (10.50 ± 2.20) compared to controls (8.70 ± 2.00), reflecting greater mobility impairment, again with a highly significant p-value (<0.001) and a large effect size (Cohen’s d = 9.85).

**TABLE 2:** Limits of stability and functional mobility in elderly individuals with hip osteoarthritis vs. age-matched asymptomatic controls.

	Measurement	Group (hip OA)	Group (controls)	p-value	Cohen’s d	Effect size (η^2^)
Limits of Stability (%)	Forward direction	41.20 ± 4.50	78.00 ± 9.00	<0.001	0.28	−3.30
	Right-Forward direction	68.00 ± 8.00	88.00 ± 11.00	<0.001	0.50	−1.84
	Right	72.00 ± 11.00	92.00 ± 12.00	<0.001	0.40	−1.76
	Right-Backward	89.00 ± 13.00	97.00 ± 14.00	<0.001	0.13	−0.58
	Backward	87.00 ± 12.00	95.00 ± 11.00	<0.001	0.20	−0.65
	Left-Backward	79.00 ± 10.00	90.00 ± 11.00	<0.001	0.53	−1.24
	Left	84.00 ± 10.00	94.00 ± 13.00	<0.001	0.27	−0.92
	Left-Forward	88.00 ± 11.00	97.00 ± 14.00	<0.001	0.21	−0.69
	Total Objective	78.00 ± 10.00	96.00 ± 12.00	<0.001	0.49	−1.47
Functional Balance Test	TUG Score	10.50 ± 2.20	8.70 ± 2.00	<0.001	9.85	0.80

Hip OA, hip osteoarthritis; p-value, Probability Value; Cohen’s d:, Cohen’s Effect Size; η^2^, Eta Squared; TUG, timed up and go.

**FIGURE 2:**
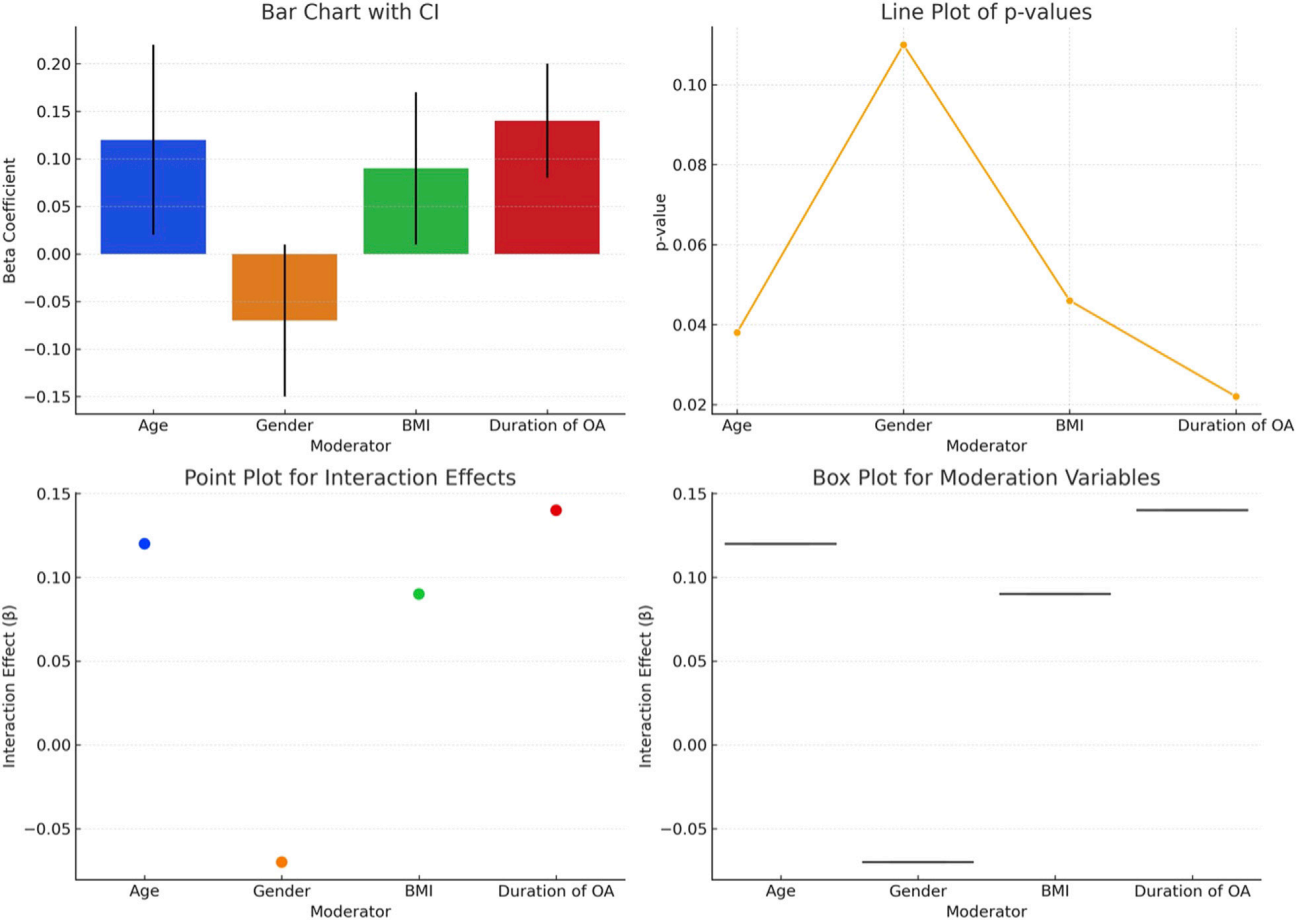
Association between stability limits and functional mobility in elderly individuals with hip osteoarthritis.

The analysis detailed in Table 3 demonstrates a significant correlation between stability limits and functional mobility in elderly individuals with hip osteoarthritis (Figure 2). Positive correlations are evident across various directions, indicating that as stability increases, so does functional mobility. The strongest positive correlation is observed in the total objective measure (r = 0.50, p = 0.009), suggesting that overall stability is a strong predictor of better functional mobility outcomes. Notably, the TUG Score, which inversely measures mobility (lower scores indicate better mobility), shows a significant negative correlation (r = −0.45, p = 0.022) with stability limits, reinforcing the relationship between increased stability and enhanced mobility capabilities. Each directional measurement, from forward to left-forward, displays moderate positive correlations, with p-values ranging from 0.018 to 0.049, underscoring the consistent impact of stability on mobility across different axes of movement.

**TABLE 3:** Association between stability limits and functional mobility in elderly individuals with hip osteoarthritis.

Measurement	Correlation coefficient (r)	p-value
Forward direction	0.45	0.023
Right-Forward direction	0.40	0.037
Right	0.38	0.045
Right-Backward	0.35	0.049
Backward	0.48	0.018
Left-Backward	0.42	0.032
Left	0.39	0.041
Left-Forward	0.41	0.035
Total Objective	0.50	0.009
TUG Score	−0.45	0.022

r, Correlation Coefficient; p, Probability Value; TUG, timed up and go.

**FIGURE 3:**
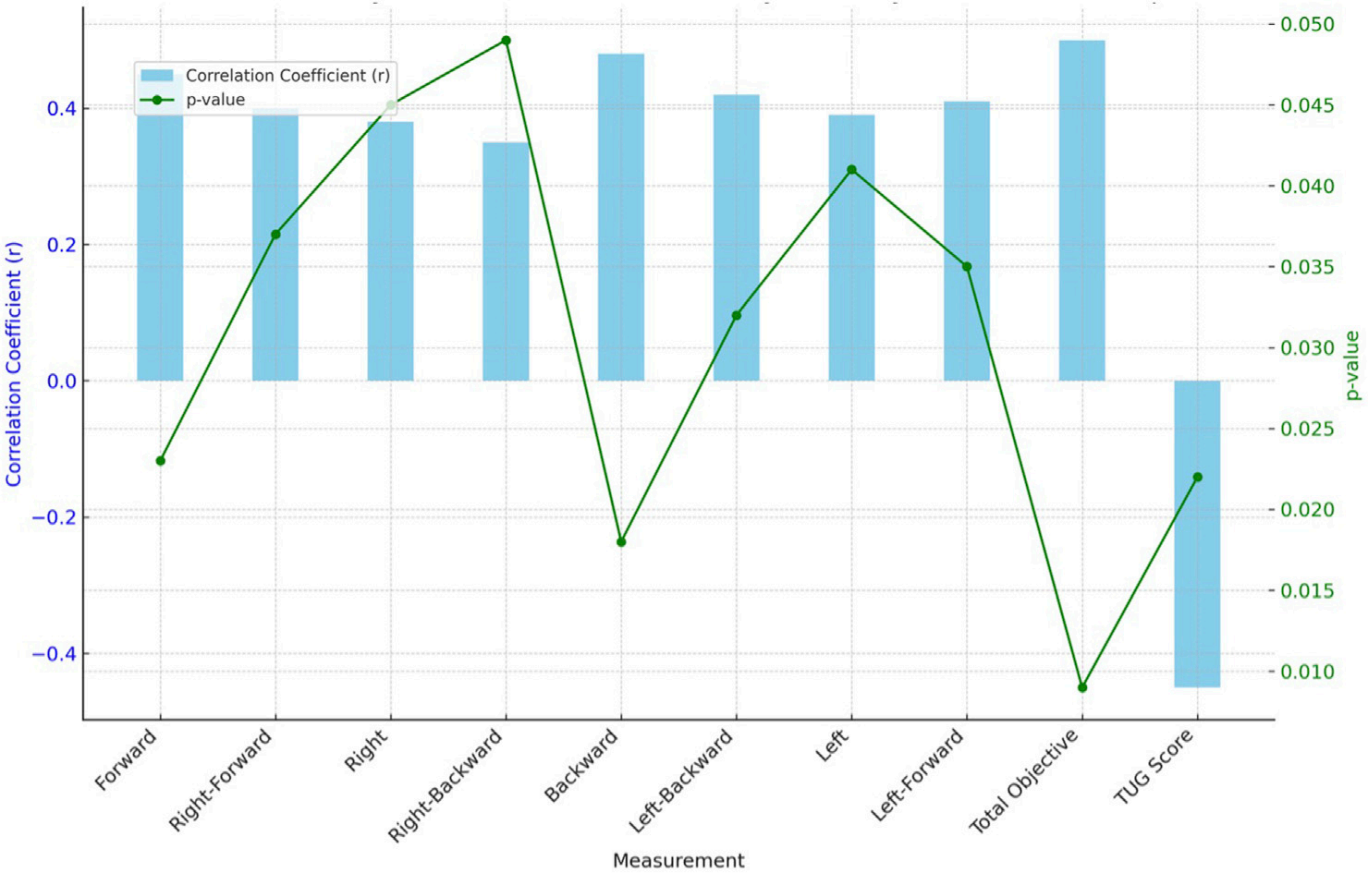
Moderation analysis of demographic and clinical variables affecting stability and mobility in elderly individuals with hip osteoarthritis.

The moderation analysis in Table 4 reveals how demographic and clinical variables influence stability and mobility in elderly individuals with hip osteoarthritis (Figure 3). Age and duration of osteoarthritis diagnosis are significant moderators in this relationship. Specifically, older age shows a positive interaction effect on mobility and stability (β = 0.12, p = 0.038), indicating that as age increases, the positive impact on the association between stability and mobility also increases. Similarly, a longer duration of osteoarthritis diagnosis significantly strengthens the relationship (β = 0.14, p = 0.022), suggesting that those who have lived longer with the diagnosis experience greater effects on their mobility and stability. In contrast, BMI shows a moderate positive influence (β = 0.09, p = 0.046), while gender does not significantly moderate the relationship (β = −0.07, p = 0.110), indicating no statistically significant differences between males and females. Although the hip osteoarthritis group had a significantly higher BMI compared to the control group, moderation analysis demonstrated that BMI had a moderate influence on the relationship between stability and functional mobility (β = 0.09, p = 0.046). However, the primary driver of balance and mobility impairments was the presence of osteoarthritis, indicating that while BMI contributed to the outcomes, it did not solely account for the observed differences.

**TABLE 4:** Moderation analysis of demographic and clinical variables on stability and mobility in elderly individuals with hip osteoarthritis.

Variable	Moderator	Interaction effect (β)	p-value	Standard error	95% CI lower	95% CI upper	Comments
Stability vs. Mobility	Age	0.12	0.038	0.05	0.02	0.22	Age moderates the relationship positively; older age strengthens the association.
Stability vs. Mobility	Gender	−0.07	0.110	0.04	−0.15	0.01	Gender has a non-significant moderating effect; difference between males and females not statistically significant.
Stability vs. Mobility	BMI	0.09	0.046	0.04	0.01	0.17	BMI moderately influences the association; higher BMI strengthens the relationship.
Stability vs. Mobility	Duration of OA	0.14	0.022	0.03	0.08	0.20	Duration of osteoarthritis diagnosis strongly moderates the relationship; longer duration increases the impact on mobility.

β, Beta Coefficient; p, Probability Value; CI, confidence interval; BMI, body mass index; OA, osteoarthritis.

## 4 Discussion

This study investigated the impact of hip osteoarthritis on stability and functional mobility among elderly patients. Significant differences were identified in both stability and mobility between patients with osteoarthritis and age-matched asymptomatic controls, with those suffering from osteoarthritis showing reduced stability and impaired mobility. Analyses demonstrated a positive correlation between stability limits and functional mobility within the osteoarthritis group, suggesting that enhanced stability is associated with improved mobility. Furthermore, moderation analysis indicated that age and the duration of osteoarthritis diagnosis significantly influence the relationship between stability and mobility, where increased age and longer disease duration exacerbate mobility limitations. These results underscore the need for tailored interventions that consider specific demographic and clinical characteristics to improve mobility and stability in elderly individuals with hip osteoarthritis.

The observed discrepancies in stability and functional mobility between elderly individuals with hip osteoarthritis and age-matched asymptomatic controls can be attributed to the mechanical and neurological impairments that osteoarthritis induces (Steinhilber, 2012). Osteoarthritis is associated with joint degradation and inflammation, which lead to pain and muscular weakness, particularly affecting the stability of the lower extremities (Shorter et al., 2019). This degeneration and the accompanying pain reduce an individual’s ability to maintain balance and perform mobility-related tasks efficiently, as demonstrated by the marked reduction in stability across all directions and the slower TUG scores (Moutzouri, 2019). The greatest disparity observed in the forward direction might be due to the forward movement engaging major hip stabilizers, which are likely compromised in patients with hip osteoarthritis (Alkhamis et al., 2024). Additionally, the deterioration of proprioceptive feedback mechanisms, which are crucial for maintaining balance and coordinating movement, further exacerbates stability issues, leading to an overall decline in functional mobility (Henry and Baudry, 2019).

Previous studies corroborate these findings, highlighting the role of muscular weakness and joint instability in decreasing mobility (Ramirez et al., 2013; Bateni and Maki, 2005). According to Meyer et al. (2018), hip osteoarthritis significantly affects the hip abductors and flexors, which are essential for lateral and forward stability, respectively. Furthermore, research by Wojcieszek et al. (2022) emphasizes that chronic pain, a common symptom in osteoarthritis patients, directly correlates with reduced mobility and poorer quality of life, as pain limits the range of motion and the willingness to engage in physical activity (Wojcieszek et al., 2022). The work by Al-Mahrouqi et al. (2020) also supports the concept that a longer duration of osteoarthritis is associated with increased severity of mobility impairments, consistent with the moderation analysis results which indicated that the duration of osteoarthritis diagnosis influences stability and mobility outcomes (Al-Mahrouqi et al., 2020). Collectively, these studies validate the observed patterns in the current research, underscoring the profound impact of osteoarthritis on stability and functional mobility, and stressing the importance of early intervention to manage symptoms and enhance life quality for affected individuals (Al-Mahrouqi et al., 2020).

The significant correlation between stability limits and functional mobility in elderly individuals with hip osteoarthritis, as detailed in the analysis, can be attributed to the integral role that musculoskeletal stability plays in enabling effective and safe movement (Raizah et al., 2023). Increased stability, particularly in those suffering from joint degenerative conditions like osteoarthritis, helps in maintaining posture and balance which are crucial for functional mobility (Sziver et al., 2016). The observed correlations suggest that improvements in stability through therapeutic interventions might enhance the capacity for movement and reduce the risk of falls (Picorelli et al., 2018). The substantial positive correlations across various directions of stability measurement, especially in the total objective, highlight that overall stability is not merely about managing individual movements but is central to enhancing holistic mobility functions (Cook et al., 2014). Conversely, the TUG Score, reflecting inverse mobility (where lower scores indicate better mobility), aligns with these findings by showing a notable negative correlation with stability limits, confirming that increased bodily stability tends to improve mobility outcomes substantially (Clemens, 2017).

Supporting these observations, previous research underscores the connection between stability and functional mobility. For instance, studies by Hirase et al. (2020) have shown that joint stability is critically important for maintaining mobility in elderly individuals, particularly those with degenerative joint diseases. Similarly, Hoglund et al. (2018) reported that targeted interventions aiming to enhance musculoskeletal stability could lead to significant improvements in the overall functional mobility of patients with hip osteoarthritis. These studies corroborate the current findings, reinforcing the critical link between enhanced stability and improved functional mobility (Hoglund et al., 2018). Furthermore, the research by Calabrò et al. (2022) demonstrates that rehabilitative strategies focused on strengthening stabilizing muscles around the hip joint not only improve stability but also contribute positively to mobility outcomes, which is consistent with the negative correlation observed between the TUG Scores and stability limits in this study. This body of evidence supports the hypothesis that improving stability through specific therapeutic approaches can substantially enhance the mobility and quality of life for elderly individuals suffering from hip osteoarthritis (Atukorala and Hunter, 2023).

The moderation analysis underscores the complex interplay between demographic and clinical variables and their impact on stability and mobility among elderly individuals with hip osteoarthritis. Age and the duration of osteoarthritis diagnosis emerge as significant moderators, enhancing the association between stability and mobility. This suggests that as individuals age or live longer with osteoarthritis, their mobility and stability are increasingly affected, likely due to the progressive nature of joint degeneration which exacerbates functional decline (Ishijima et al., 2016). In contrast, while body mass index (BMI) also shows a moderate positive influence, indicating that higher BMI might exacerbate mobility issues, gender does not significantly alter this relationship (Kennedy et al., 1997). These findings align with previous research, such as that by Kadam and Croft (2007), who noted that increased age and chronicity of symptoms typically worsen mobility outcomes in osteoarthritic patients. Furthermore, studies by Chen et al. (2020) support the notion that higher BMI contributes to poorer joint function and mobility due to greater mechanical load on compromised joints. The absence of a significant gender effect might suggest that both sexes are equally susceptible to the biomechanical and symptomatic impacts of osteoarthritis on mobility and stability, which contrasts with some literature suggesting variations in disease impact and coping mechanisms between genders (Vincent and Mathews, 2013). This comprehensive analysis not only corroborates existing theories about osteoarthritis progression but also highlights the critical need for personalized management strategies that address these specific factors to optimize mobility and quality of life in this population (Maly et al., 2020).

This study, while providing valuable insights into the stability and mobility impairments in elderly individuals with hip osteoarthritis, is not without limitations. One of the primary constraints is the cross-sectional design, which, although effective for identifying associations at a specific point in time, does not allow for the determination of causality between osteoarthritis progression and mobility impairments. Additionally, the study relied on self-reported data for the duration of osteoarthritis diagnosis, which could introduce recall bias affecting the accuracy of the reported disease duration. Furthermore, the sample size, while adequate for detecting moderate effects, may not fully capture the variability and nuances of more subtle clinical differences within the population, particularly when analyzing the effects of demographic variables such as gender. The study also did not account for potential confounders like activity level or other comorbidities, which could influence mobility and stability independently of osteoarthritis. Future studies could benefit from a longitudinal design, larger and more diverse samples, and more comprehensive data collection strategies to address these limitations and enhance the understanding of the dynamics between osteoarthritis and functional mobility.

## 5 Conclusion

The study conclusively demonstrates that elderly individuals with hip osteoarthritis experience significant impairments in stability and functional mobility compared to age-matched asymptomatic controls, with stability strongly correlating with mobility outcomes. Moderation analysis further revealed that age and duration of osteoarthritis significantly influence the interplay between stability and mobility, suggesting that longer disease duration and advanced age exacerbate these impairments. Notably, body mass index also moderately affects mobility, whereas gender does not significantly impact the observed associations. These findings highlight the profound impact of hip osteoarthritis on the functional capabilities of the elderly, underscoring the necessity for targeted therapeutic interventions that prioritize stability enhancement to improve mobility and overall quality of life in this population. This research adds to the body of knowledge by quantitatively establishing the relationships between biomechanical stability, demographic factors, and mobility in individuals burdened by osteoarthritis, paving the way for more tailored and effective clinical management strategies.

## Data Availability

The datasets presented in this study can be found in online repositories. The names of the repository/repositories and accession number(s) can be found below: https://doi.org/10.5281/zenodo.11382872.
